# Bicyclist-evoked arousal and greater attention to bicyclists independently promote safer driving

**DOI:** 10.1186/s41235-021-00332-y

**Published:** 2021-10-21

**Authors:** Andy Jeesu Kim, Hananeh Alambeigi, Tara Goddard, Anthony D. McDonald, Brian A. Anderson

**Affiliations:** 1 Department of Psychological & Brain Sciences, Texas A&M Institute for Neuroscience, Texas A&M University, 4235 TAMU, College Station, TX 77843-4235 USA; 2 Present Address: University of Southern California, Los Angeles, USA; 3 Industrial & Systems Engineering, Texas A&M University, 3127 TAMU, College Station, TX 77843-4235 USA; 4 Landscape Architecture & Urban Planning, Texas A&M University, 3127 TAMU, College Station, TX 77843-4235 USA

**Keywords:** Attentional bias, Arousal, Anxiety, Driving simulator

## Abstract

While attention has consistently been shown to be biased toward threatening objects in experimental settings, our understanding of how attention is modulated when the observer is in an anxious or aroused state and how this ultimately affects behavior is limited. In real-world environments, automobile drivers can sometimes carry negative perceptions toward bicyclists that share the road. It is unclear whether bicyclist encounters on a roadway lead to physiological changes and attentional biases that ultimately influence driving behavior. Here, we examined whether participants in a high-fidelity driving simulator exhibited an arousal response in the presence of a bicyclist and how this modulated eye movements and driving behavior. We hypothesized that bicyclists would evoke a robust arousal and orienting response, the strength of which would be associated with safer driving behavior. The results revealed that encountering a bicyclist evoked negative arousal by both self-report and physiological measures. Physiological and eye-tracking measures were themselves unrelated, however, being independently associated with safer driving behavior. Our findings offer a real-world demonstration of how arousal and attentional prioritization can lead to adaptive behavior.

## Significance statement

The influence of negative arousal on attentional biases and behavior has been consistently observed in laboratory tasks, but the functional consequences of such biases in real-world situations are largely unexplored. Drivers often view bicyclists as unpredictable and carry negative perceptions toward bicyclists, with potential consequences for driving behavior, offering a real-world context within which to examine this issue. In this study, using a high-fidelity driving stimulator, we examined the relationship between self-reported anxiety and arousal evoked by bicyclists, visual attention to bicyclists, and behaviors related to safe driving. Physiological and eye-tracking measures suggested that negative arousal and attentional bias toward bicyclists are independently associated with safer driving behaviors and can be functionally adaptive.

## Introduction

The world is filled with vast amounts of information in a dynamically changing environment. Attention is the cognitive process that selectively filters incoming sensory information to determine which stimuli are ultimately represented in the brain (Desimone & Duncan, 1995). Attention can be voluntarily directed to or reflexively oriented toward features and spatial locations that are in accordance with our goals (e.g., Abrams et al., 2010; Corbetta & Shulman, 2002; Corbetta et al., 2000; Duncan & Humphreys, 1989; Folk et al., 1992; Gabay & Henik, 2010; Posner, 1980; Wolfe, 1994). In addition, the physical salience of stimuli has been shown to automatically direct attention (e.g., Kahneman et al., 1992; Pashler, 1988; Theeuwes, 1991, 1992, 2010). More recently, learned associations between stimuli and reward (e.g., Anderson et al., 2011; Della Libera & Chelazzi, 2006; Engelmann & Pessoa, 2007; Kiss et al., 2009; Navalpakkam et al., 2010) and punishment (e.g., Chubala & Smith, 2009; Koster et al., 2004; Schmidt et al., 2015a, 2015b), along with implicit learning of statistical relationships among sensory cues (e.g., Chun & Jiang, 1998; Fiser & Aslin, 2001; Frost et al., 2015; Turk-Browne et al., 2005), have been shown to influence the control of attention.

How negatively valenced stimuli are processed by the attention system has been an area of longstanding interest, examined most prominently in the context of attention to stimuli that evoke a sense of immediate threat. Experimental paradigms have consistently demonstrated that attention is biased toward fearful or threatening stimuli using fearful faces (e.g., Dimberg & Ohman, 1996; Eastwood et al., 2001; Eldar et al, 2010; Vuilleumier, 2005), threatening animals (e.g., snakes, spiders; Ohman & Mineka, 2003; Ohman et al., 2001), negative-valence images (e.g., Derryberry & Reed, 2002; Most et al., 2005; Quigley et al., 2012), or threatening words (e.g., Mathews & Macleod, 1985, 1994). Participants have also demonstrated longer dwell times on threatening stimuli compared to neutral stimuli, providing evidence for delayed disengagement from threat-associated stimuli (e.g., Fox et al., 2002; Georgiou et al., 2005; Grafton et al., 2012). In addition, learned associations between arbitrary stimuli and aversive outcomes have also been shown to automatically influence the control of attention, as seen with electric shock and aversive white noise (e.g., Anderson & Britton, 2020; Chubala & Smith, 2009; Koster et al., 2004; Schmidt et al., 2015a, 2015b). These findings demonstrate that the attention system is not only biased to innately threatening objects, but also prioritizes objects that poses a learned threat.

In contrast to such investigations of attentional biases toward threat, relatively few studies have investigated changes in attentional processing when the observer is in an anxious or negatively aroused state. Using self-report measures of state anxiety, highly anxious participants have shown increased attentional biases toward physically salient stimuli (e.g., Esterman et al., 2013; Moser et al., 2012). Furthermore, the threat of unpredictable electric shock has been shown to induce negative arousal, which elevates the attentional priority afforded to physically salient stimuli and reduces the attentional priority afforded to previously reward-associated stimuli (Kim & Anderson, 2020a, b; see also Mather & Sutherland, 2011; Sutherland & Mather, 2012, 2015). Furthermore, a state of negative arousal induced by a threat-of-shock manipulation has been shown to facilitate goal-directed attentional control and improve task performance (Kim et al., 2021). However, the functional significance of negative arousal with respect to attention and behavioral performance in everyday life situations remains poorly characterized.

Automobile driving poses an interesting test case for the real-world consequences of negative arousal and how it affects attention and behavior. Drivers can have negative perceptions toward bicyclists due to their unpredictability (e.g., Goddard et al., 2016; Johnson et al., 2014) and have also reported feeling nervous and startled by a bicyclist’s presence (Goddard, 2017; Goddard et al., 2020). In addition, drivers have perceived bicyclists as competition for limited roadway space and hold negative views of bicyclist’s rule-following, courteousness, and responsible roadway behavior (e.g., Gatersleben & Haddad, 2010; Goddard et al., 2016; Goddard, 2016, 2017; Johnson et al., 2014). A failure to detect the danger posed by a potential hazard is a leading cause of crashes (Herslund and Jørgensen, 2003), while at the same time, anxiety is associated with elevated attention to threatening stimuli (see Bar-Haim et al., 2007, for a meta-analysis) and more unsafe driving performance (e.g., Fairclough et al., 2006; Matthews et al., 1998; Taylor et al., 2007; Wilson et al., 2006; Wong et al., 2015).

Driving simulators have been utilized to emulate real-world scenarios for drivers and their interactions (Fisher et al., 2011; see also Wynne et al., 2019, for a review). In addition to collecting driving data, researchers conducting driving simulator experiments have explored driver stress by observing changes in physiological indicators such as electrodermal activity (EDA, e.g., Daviaux et al., 2020; Kajiwara, 2014; Paschalidis et al., 2018; Steinberger et al., 2017), electrocardiography (e.g., Brookhuis & De Waard, 2010; Lanatà et al., 2014; Steinberger et al., 2017), heart/breathing rates (e.g., Brookhuis & De Waard, 2010; Paschalidis et al., 2018; see also Lohani et al., 2019, for a review), facial temperature (e.g., Kajiwara, 2014), and eye movements (e.g., Ebadi et al., 2020; Körber et al., 2015; Merat & Jamson, 2013). Bicyclists have been integrated into driving simulator studies to investigate and resolve vehicle–bicyclist conflicts (e.g., Ebadi et al., 2020; Farah et al., 2019; Goddard, et al., 2020; Hou et al., 2019; O’Hern et al., 2019). While prior findings demonstrate that drivers perceive bicyclists in a negative light and as potentially threatening (e.g., Gatersleben & Haddad, 2010; Goddard, 2016, 2017; Goddard et al., 2016; Johnson et al., 2014), it is unclear whether encountering bicyclists while driving is associated with physiological changes and whether such changes are associated with attentional biases and ultimately modulate driving behavior.

In the present study, we examined whether encountering a bicyclist while driving is associated with an arousal response and corresponding attention, and whether more pronounced arousal and attentional improve or hinder driving behavior relevant to interactions with a bicyclist (measured from distance between driver and bicyclist, decisions whether and when to pass a bicyclist, and consistency of lane position during bicyclist interaction). First, we evaluated the degree to which bicyclists evoked arousal by measuring changes in physiological responses when the bicyclist appeared in the driver’s field of view, and the degree to which the driver’s attention was biased toward the bicyclist by measuring eye movements. If the bicyclist does evoke an arousal response, it is unclear whether the magnitude of this response would be associated with closer encounters with bicyclists and more inconsistent control of the vehicle or instead facilitate greater distance between the driver and bicyclist and more consistent lane position, which are related to bicyclist safety. Likewise, greater attention to bicyclists could benefit driving performance (more careful tracking and avoidance) or hinder it (insufficient attention to lane position and other roadway events and/or a tendency to drift toward the point of fixation when looking at the bicyclist). Although measured physiological arousal is necessarily ambiguous with respect to valence, we interpret bicyclist-evoked arousal in our experiment as negative given the prior literature concerning bicyclist–driver interactions (e.g., Goddard, 2017; Goddard et al., 2016, 2020; Johnson et al., 2014) and self-reported anxiety concerning these interactions obtained in a related study examining driver attitudes toward bicyclists and bicyclist encounters in the same sample of participants tested here (Goddard et al., 2020). We hypothesized that bicyclists would evoke a robust arousal and orienting response, the strength of which would be positively correlated with each other and with safer driving behavior (due to enhanced attentional processing of, and greater concern for, the bicyclist).

## Materials and methods

### Participants

One hundred and five participants (female = 66, male = 38, no response = 1) whose ages ranged from 18 to 54 years (*M* = 26.7, SD = 9.9) were recruited from the Texas A&M University community. All participants were English-speaking, reported normal or corrected-to-normal visual acuity and normal color vision, held a valid driver’s license, reported driving experience of at least 1.5 years, and were not on any medications that may have affected the operation of a moving vehicle. All procedures were approved by the Texas A&M Institutional Review Board (IRB2018-0659D) and were conducted in accordance with the principles expressed in the Declaration of Helsinki. Written informed consent was obtained from each participant. Participants received monetary compensation of $10/h. No participants withdrew early from the study due to simulator sickness, and all participants completed the entire experiment.

### Apparatus

**Fig. 1:**
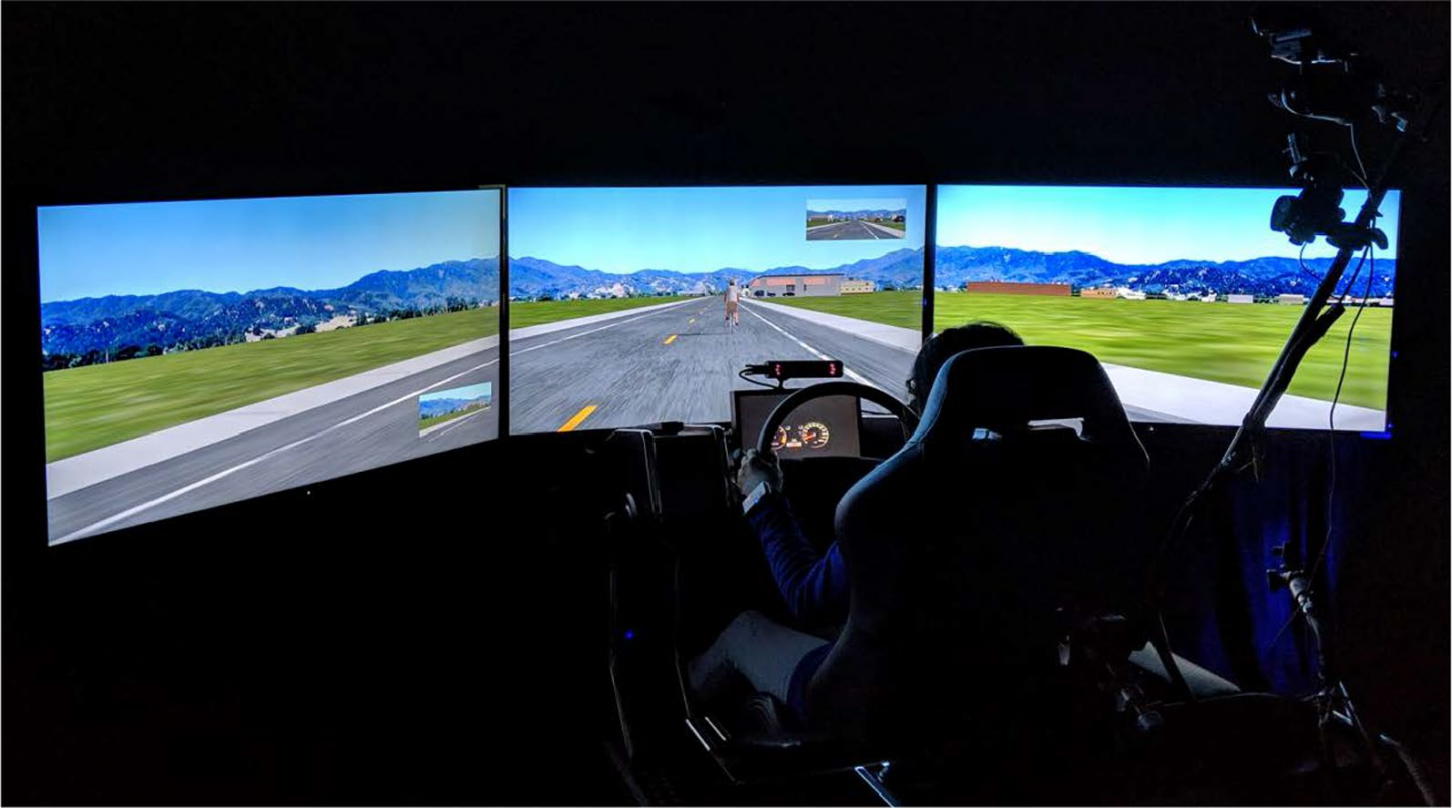
Driving simulator apparatus. *This figure was published in Goddard et al. (2020), Copyright Elsevier 2020. Reprinted with permission

This study was conducted in the Texas A&M Transportation Institute’s Realtime Technologies Inc. driving simulation environment. The high-fidelity driving simulator consisted of a single-seat, three screens positioned panoramically (subtending 165° visual angle horizontally and 35° vertically), and a speaker system to provide ambient roadway noise (Fig. 1; see Goddard et al., 2020, for a detailed description). Drivers controlled their virtual vehicle through a force feedback steering wheel, accelerator, and a brake pedal. The driving environment and vehicle responses were simulated using the SimCreator software. Driver performance data were collected during two experimental drives, including steering wheel position, accelerator pedal position, brake pedal position, velocity, time to lane crossing, time headway to an upstream object, and lane position at a 10 Hz sampling rate.

Throughout the experiment, physiological indicators from each participant were measured including heart rate, breathing rate, and EDA. Heart rate and breathing rate were measured using the Zephyr BioHarness 3.0 (Zephyr Technology, Annapolis, MD, USA). The sensor was connected to a chest strap that was worn underneath the subject’s clothing to maintain skin contact. The BioHarness 3.0 can detect heart rate in the range of 25–240 bpm and breathing rate in the range of 4–70 bpm. In addition, EDA was measured using the Shimmer3 GSR sensor (Shimmer, Dublin, Ireland) from the palm. The Shimmer3 sensor can measure EDA in the range of 10–4700 kΩ.

Eye movements were captured in the experimental drives using a 60 Hz single-camera FOVIO system (Seeing Machines Inc., Canberra, Australia). The FOVIO system was interfaced with the Eyeworks Data Record software (Eyetracking Inc., Solana Beach, CA, USA), and movie files of a moving dot (denoting where the participant was looking) were saved using a script from EyeWorks Design (Eyetracking Inc.). Participants were calibrated to the FOVIO system using a four-point calibration screen and were instructed to look at the exterior edges of the panoramic display while maintaining a directly forward field of view.

### Experiment procedure

After completing the consent forms and demographic surveys, participants were connected to the EDA and BioHarness monitors. The participants were then seated on the driving simulator and monitored for stable connectivity with live readings from both physiological monitors. Lastly, participants were calibrated to the FOVIO eye-tracking camera. All participants completed three virtual drives: one practice and two experimental drives. The goals of the practice drive were to familiarize the participants with the simulator controls and screen participants who experienced severe simulator sickness. The two experiment drives differed in the posted speed limit: 40 mph (64.37 kph) in the first drive and 25 mph (40.23 kph) in the second drive. Before the start of each drive, the experimenter told the participant the speed limit of each drive, and the virtual world contained speed limit signs before and after every programmed vehicle–bicyclist interaction (described below). Since interactions were triggered based on distance from the bicyclist, the experimenter verbally instructed the participant to monitor their speed if they exceeded the speed limit. The total length of the experiment took approximately 1 h.

The simulated driving environment and scenarios are described in detail in Goddard et al. (2020). In brief, four interactions with the bicyclist were programmed into the simulator scenarios: overtake, right turn, left turn, and intersection. During the overtake event, participants encountered a bicyclist riding inside the lane in front of the vehicle on the roadway and were forced to decide when to overtake the bicyclist or to continue trailing behind the bicyclist (see Fig. 1). During the right turn event, participants again encountered a bicyclist riding inside the lane in front of the vehicle while approaching a right turn intersection and were forced to decide to pass the bicyclist to turn right before the intersection or to wait for the bicyclist to pass the coming intersection first before turning. During the left turn event, participants encountered a bicyclist coming toward them on the opposite side of the road and were forced to decide whether to turn left in front of the bicyclist before it arrived at the intersection or to stop and wait for the bicyclist to pass through the intersection first before turning. During the intersection event, participants encountered a bicyclist driving toward a four-way intersection, perpendicular to the driver from the right side. The event was programmed so that the bicyclist and driver would arrive at approximately the same time at the intersection to give participants the option to wait and let the bicyclist go through first or to cross through the intersection first while the bicyclist is stopped.

Following the two experiment drives, participants were disconnected from the EDA and BioHarness sensors. Lastly, participants were relocated to a separate room where completed a short survey before completing the experiment.

### Self-report survey

Participants completed a self-report survey probing explicit attitudes toward bicyclists, which was previously described in Goddard et al. (2020). Here, we focused on items relevant to threat orientation toward the bicyclist and potential for adaptive driving behavior. Specifically, we included the survey questions “It makes me nervous when I have to drive near a bicyclist,” “It has startled me when a bicyclist comes up on my driver’s side,” “If I don’t pass a bicyclist, other drivers get angry,” and “I am a skilled driver.” Responses were recorded on a six-point Likert-scale (Strongly Disagree to Strongly Agree).

### Additional measures

As a part of a different study focused on relating driver attitudes to driver–bicyclist interactions, participants completed an implicit association test (IAT), which was previously developed to assess subconscious attitudes concerning drivers and bicyclists (Goddard, 2017). We did not have any specific a priori hypotheses in regard to the IAT data with respect to the present study, and so we did not include this measure in our analyses; analyses linking implicit and a broader range of explicit driver attitudes to overtaking behavior specifically are reported in Goddard et al. (2020).

### Data preprocessing and analyses

#### Eye-tracking data

The movie files exported from the eye-tracking software were used to quantify saccades made to, and the fixation duration on, three categories of interest: the road and other vehicles, the bicyclist, and the side/rear-view mirrors (along with other task-irrelevant objects on the screen). Two different individuals manually coded the videos by counting the saccades to and the fixation duration on (using the elapsed time in the movie files) these three categories of objects during each interaction. We defined the start of each interaction as when the bicyclist first becomes visible on-screen and the end of each interaction as after the driver passes the bicyclist and it is no longer in the participant’s field of view. For the overtake, right turn, and left turn events, the bicyclist was defined as visible when the participant was driving toward the bicyclist and was 225 m away, while the end of the event was defined as when the driver had passed the bicyclist and was 25 m away. For the intersection event, the bicyclist became visible to the participant when the driver was 70 m away from the bicycle and the end of the event was defined as when the bicyclist had gone through the intersection and was 15 m away from the driver.

To verify the consistency of the data produced by the two coders, we calculated the inter-rater reliability by correlating their scores for each measure. The raw number of saccades to and fixation duration on each measure was z-scored for each coder. In addition, to account for human error, a third individual acted as an “arbitrator” when there was substantive disagreement among the coders. We calculated the difference in z-scores between the two coders for each measure and determined that the third individual would code measures that differed by more than 2 z-score units. For these instances, the two data points that were closest in proximity (out of the three individual coders) were chosen as the final data points. The inter-rater reliability between the two coders for all measures was *rs* > 0.71 (averaged across all measures *r* = 0.79).

To account for individual differences in overall eye movements made between participants (which was influenced by the quality of eye tracking and the number samples in which the pupil was lost for each participant), the saccades to and fixation duration on the bicyclist were analyzed as a proportion of all valid gaze data, as a measure of attentional bias toward the bicyclist. Lastly, we calculated the time to fixate the bicyclist on the first overtake interaction during the first experimental drive as a proxy measure for orienting time to the bicyclist when it is first introduced to the driver (i.e., before the driver knows that bicyclists can appear in the simulation and might start to overtly monitor for them).

#### Physiological data

For all physiological measures (heart rate, breathing rate, and EDA), we focused on two time periods of interest: baseline and when visually encountering the bicyclist. Baseline time periods were defined as the 12 s time period of free driving in the simulator world before seeing the bicyclist. The “encountering bicyclist” time period was defined as the 12 s time period after the bicyclist became visible. Given the inherent variability in the temporal unfolding of the driver–bicyclist interaction and potential variability in the resulting time course of physiological responses, we established a standardized time window of 12 s a priori to allow for ample time for capturing physiological changes and to not overlap with other events in the drive. In addition, we focused analyses on the peak response to better account for variability in the total duration of the bicyclist interaction and individual differences in time to notice the bicyclist. All EDA responses were preprocessed using the AcqKnowledge 5.0 software (BIOPAC Systems Inc., Goleta, CA, USA). To analyze event-related skin conductance responses (ER-SCR) elicited when seeing the bicyclist, phasic EDA responses were derived by delineating the presence of SCRs within the tonic signal. All preprocessing steps used the default AcqKnowledge 5.0 software parameters and guidelines recommended in Braithwaite and Watson (2015).

### Correlation matrix

We calculated a correlation matrix consisting of the physiological, eye-tracking, driving, and self-report measures and conducted a randomization test to quantify the evidence for or against our aforementioned hypothesis linking arousal (and self-reported emotion), eye movements, and driving behavior. For the randomization test, correlations that were predicted to be negative were sign-flipped such that the strength and direction of each correlation corresponded to the strength and direction of evidence for or against our hypothesis. Then, the sign of each correlation was randomly flipped through 100,000 iterations, and a distribution of the sum of the correlation coefficients was computed, against which the observed sum of the actual correlations was compared. Such a test allows assessment of the *overall pattern* of relationships among our variables and whether that pattern is more consistent with our hypothesized interrelationships among variables than would be expected by chance, providing a wholistic picture that avoids the multiple comparisons problem. This analysis accounts for both the direction and magnitude of individual correlations, and whether the sum total of the correspondence between these correlations and the hypothesized relationships is greater than would be expected under the null hypotheses that our variables were not related to each other.

We included all three of our physiological measures (heart rate [HR], breathing rate [BR], and electrodermal activity [EDA]), all three of our eye-tracking measures (time to fixate bicyclist [Time Fix], saccades to bicyclist [Sacc Bike], and fixation duration on bicyclist [Dur Bike]), all driving measures related to safe driving behavior (minimum distance from bicyclist during overtake interaction [Dist Over], minimum distance from bicyclist during the right, left, and intersection interactions, averaged over the three interactions [Dist Avg], decision whether or not to pass the bicyclist during overtake interaction [Pass Bike], decision to pass and turn in front of the bicyclist during the right turn interaction [Turn Bike], and standard deviation of lane position during the overtake interaction [Lane Pos]), and self-report measures that could influence perceiving the bicyclist as a threat and self-evaluated driving skill (“It makes me nervous when I have to drive near a bicyclist” [Nervous], “It has startled me when a bicyclist comes up on my driver’s side” [Startled], “If I don’t pass a bicyclist, other drivers get angry” [Pressure], and “I am a skilled driver” [Skilled]). When including the distance from bicyclist measure in our correlation matrix, we separated the measure into the overtake interaction and the average distance over the other three interactions (right, left, and intersection) because the overtaking maneuver is unique with respect to the other types of maneuvers concerning intersections, particularly in that it necessitates a close encounter (see, e.g., Goddard et al., 2020). All measures to be included in the correlation matrix and the hypothesized nature of the relationships were determined a priori.

### Data exclusion criteria

Data from four participants were removed due to a software bug in the virtual simulation that resulted in the bicyclist interaction not unfolding as programmed (which included three participants who collided with the bicyclist, with the collision inducing a bug). Thus, driving data were analyzed from a total of one hundred and one participants. To account for movement artifacts in the physiological data due to the effect of poor fitting to the sensors and changes in posture during the experimental drives, we removed data from epochs that featured at least one value outside the valid range of measurement from both physiological sensors. Thus, heart rate and breathing rate data were analyzed from a total of 92 participants and EDA was analyzed from a total of 88 participants. Lastly, eye-movement data from six participants were unable to be collected, due to major movements of the head that the tracking software could not tolerate that resulted in an inability to maintain stable eye position. Thus, eye-movement data from a total of 95 participants were acquired and analyzed. For each individual correlation computed, all retained data points were used, with different measures having a slightly different number of data points.

## Results

**Fig. 2:**
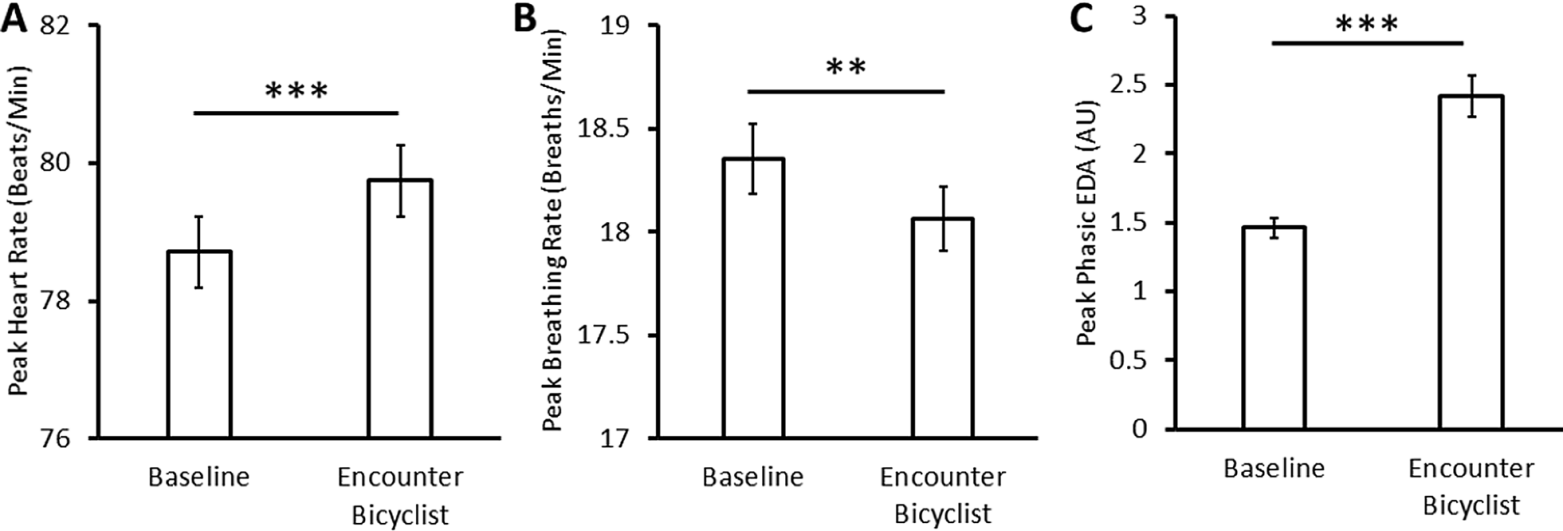
Changes in (**A**) peak heart rate, (**B**) peak breathing rate, and (**C**) peak phasic EDA in epochs when the bicyclist enters the driver’s field of view. Error bars depict within-subject confidence intervals calculated using the Cousineau method with a Morey correction. **p < 0.01. ***p < 0.001

We first evaluated whether the bicyclist evoked negative arousal by measuring physiological changes upon viewing the bicyclist compared to baseline periods of the task (Fig. 2). Participants showed significant increases in peak heart rate (baseline: *M* = 78.7, SE = 0.52; encounter bicyclist: *M* = 79.7, SE = 0.52), *t*(91) = 4.08, *p* < 0.001, *d*_*z*_ = 0.46, and EDA (baseline: *M* = 1.46, SE = 0.07; encounter bicyclist: *M* = 2.42, SE = 0.15), *t*(87) = 4.12, *p* < 0.001, *d*_*z*_ = 0.44, when the bicyclist appeared in their field of view. In contrast, participants showed significant decreases in peak breathing rate when encountering the bicyclist (baseline: *M* = 18.3, SE = 0.17; encounter bicyclist: *M* = 18.1, SE = 0.16), *t*(91) = 3.09, *p* = 0.003, *d*_*z*_ = 0.29, potentially to some degree holding their breath during the interaction.

**Fig. 3:**
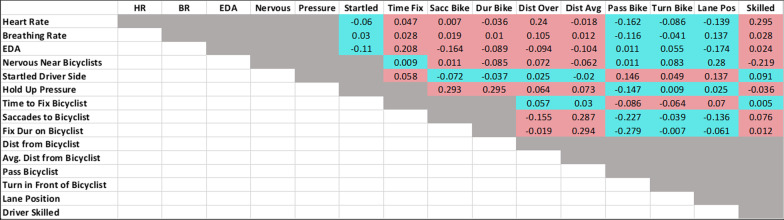
Correlation matrix containing physiological, eye-tracking, driving, and questionnaire measures, color-coded based on predictions arising from the hypothesis that anxiety promotes safer interactions with bicyclists while driving (red = positive, blue = negative)

Next, using the randomization test (see Materials and methods), we examined the relationships between the self-report responses, physiological and eye-tracking measures, and simulator behaviors that were illustrated in our correlation matrix (Fig. 3). Our correlation matrix and randomization test allowed us to investigate whether the collection of acquired measures reliably supports our aforementioned hypothesis that the bicyclist elicits a negative arousal response leading to increased attention to the bicyclist and compensatory driving behaviors. We were able to reject the null hypothesis with high confidence (*p* = 0.005); that is, the hypothesized relationship among our dependent measures was collectively stronger than would be expected by chance.

**Fig. 4:**
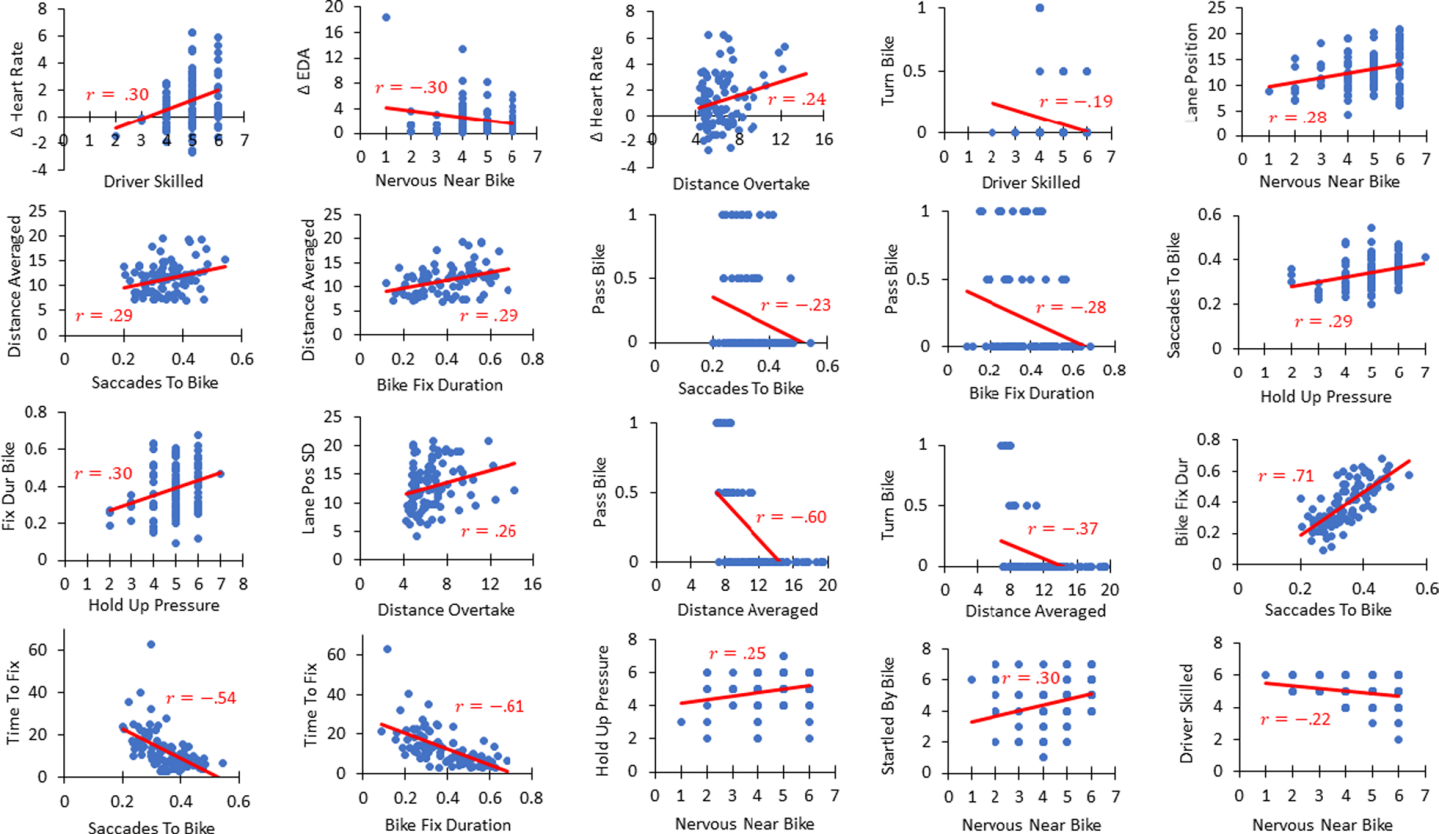
Significant relationships (uncorrected for multiple comparisons) between physiological, eye-tracking, driving, and self-report measures

Given that the collection of measures reliably supports our hypothesis, we further explored the individual relationships between measures. That is, we broke down individual correlations with respect to the aspect of our hypothesis that they speak to. This is tantamount to performing post hoc contrasts to probe a significant main effect in an analysis of variance. (All of the following p-values are uncorrected for multiple comparisons and, given the very large number of measures obtained, would in general not survive stringent multiple comparisons corrections; see Fig. 4.) Overall, 8 correlations were significant and consistent with our hypothesis and 2 correlations were significant and contrary to our hypothesis (both involving self-report concerning driving in everyday life). The following sections explore these correlations based on specific questions arising from our hypotheses.

### Is bicyclist-evoked arousal associated with safe driving behavior?

Consistent with our hypothesis, heart rate was correlated with distance between the driver and the bicyclist during the overtake maneuver (*r* = 0.240, *p* = 0.023) and self-reported driver skill (*r* = 0.295, *p* = 0.005). No other pertinent correlations were significant. Given these individual relationships and the overall pattern evident from our randomization test, our data are consistent with the idea that greater bicyclist-evoked arousal is associated with safer driving.

### Is greater attention to bicyclists associated with safe driving behavior?

Consistent with our hypothesis, proportion of saccades toward the bicyclist and fixation duration was correlated with distance from the bicyclist during the non-overtake interactions (*r* = 0.287, *p* = 0.005; *r* = 0.294, *p* = 0.004, respectively), and also with the decision to pass in front of the bicyclist (*r* =  − 0.227, *p* = 0.028; *r* =  − 0.279, *p* = 0.006, respectively). No other pertinent correlations were significant. Given these individual relationships and the overall pattern evident from our randomization test, our data are consistent with the idea that greater attention to the bicyclist is associated with safer driving.

### Are bicyclist-evoked arousal and attention to bicyclists related?

No significant correlations were observed between eye movement measures and any of our three measures of physiological arousal. Although each is individually associated with safe driving as outlined above, we find no evidence for the idea that arousal and attention to the bicyclist are related to each other; rather, they appear to independently predict driving behavior.

### Is self-reported nervousness/anxiety associated with safe driving or attention?

Consistent with our hypotheses, self-reported hold-up pressure was correlated with both the proportion of saccades toward the bicyclist and fixation duration on the bicyclist (*r* = 0.293, *p* = 0.004; *r* = 0.295, *p* = 0.004, respectively). Counter to our hypothesis, self-reported nervousness near bicyclists was positively correlated with variability (standard deviation) in lane position (*r* = 0.280, *p* = 0.004) and self-reported driver skill (*r* =  − 0.219, *p* = 0.025). These contradicting relationships offer no clear conclusions on our hypotheses.

### Relationships among other variables

We investigated whether variables within a specific category had significant relationships to provide more context to our group measures. We first evaluated whether our physiological measures were in-sync with each other. However, no significant correlations were identified, suggesting that these three measures capture different components of arousal in our task. Within the eye-movement measures, proportion of saccades toward the bicyclist was highly correlated with proportion of fixation duration on the bicyclist (*r* = 0.719, *p* < 0.001), predictable given that they are non-independent measures. However, we also investigated whether increased attentional biases as indicated by saccades and fixation duration were associated with faster first fixation time on the bicyclist. Time to first fixate the bicyclist was correlated with both the proportion of saccades toward the bicyclist and the fixation duration on the bicyclist (*r* =  − 0.540, *p* < 0.001; *r* =  − 0.611, *p* < 0.001, respectively), demonstrating a correspondence between initial orienting and sustained attentional priority. Several strong relationships were identified among driving measures, particularly those that were non-independent (e.g., distance to the bicyclist during non-overtake interactions and decisions whether to pass). Comparing self-report measures of anxiety concerning bicyclists and physiological indicators of arousal, self-reported nervousness near bicyclists was correlated with EDA (*r* =  − 0.297, *p* = 0.021). In general, there was no clear relationship between the self-report measures evaluating real-life bicyclist interactions and the magnitude of physiological arousal evoked by bicyclists in our simulated driving task. We include these significant relationships within categories of measures in Fig. 4 for completeness.

## Discussion

We hypothesized that bicyclists on the road would evoke physiological arousal during a simulated drive, based on evidence that drivers (including the participants in our sample; Goddard et al., 2020) find driver–bicyclist interactions anxiety-provoking (e.g., Johnson et al., 2014; Goddard et al., 2016; Goddard, 2017), and that arousal-induced attentional biases would promote safe driving behavior. A reliable stress response was observed using physiological indicators of arousal when the bicyclist appeared in the driver’s field of view, mimicking responses evoked by threat-associated stimuli or threatening objects in laboratory settings (e.g., Bradley et al., 2001; Eippert et al., 2007; Felmingham et al., 2011). Furthermore, our correlation matrix and randomization test based on our a priori hypothesis demonstrated that the collection of self-report, physiological, eye-tracking, and driving measures were overall consistent with the idea that greater bicyclist-evoked arousal is associated with greater attention to bicyclists, facilitating safer driving behavior. Post hoc analyses more thoroughly characterized our findings in that negative arousal, measured by both self-report and physiological measures, and attentional priority allocated to the bicyclist were associated with safer driving. Moreover, the measures of arousal and eye movements were themselves unrelated, suggesting these measures independently influence driving behavior, although one relationship between self-reported anxiety (hold-up pressure) and attention was observed.

A state of anxiety has been shown to be either adaptive or disruptive to task performance under different conditions (e.g., Cornwell et al., 2012; Grillon, 2008; Grillon & Charney, 2011; Hu et al., 2012; Lindstrom & Bohlin, 2012; Miu et al., 2008; Robinson et al., 2011, 2013; Vytal et al., 2013; Yang et al., 2018), and it is unclear whether arousal evoked by driver–bicyclist interactions is adaptive or disruptive to goal-directed behavior during these interactions. Our findings are broadly consistent with those of Kim et al. (2021) who showed that threat-induced anxiety can improve performance in a goal-directed task. Although Kim et al. (2021) utilized a computerized attention task and the present study emulated real-world conditions using a driving simulator, both studies required participants to complete a goal-directed task in a dynamically changing environment under anxiety-provoking conditions.

It is important to recognize that the bicyclist was not a physically salient object in the virtual simulator world nor did it pop-out onto the driver’s field of view. Rather, the bicyclist gradually came into view from a distance and was one of many objects visible at any one moment in the task. It thus seems unlikely that the bicyclist would have involuntarily captured attention due to considerations concerning physical salience, such that the physical properties of the bicyclist would explain why participants attended to it. Rather, the bicyclist was attended because it was recognized as a potential obstacle that was relevant to current driving considerations. At least within the confines of our virtual driving task, greater attention directed to the bicyclist was associated with safe driving behavior. It was not the case that greater attention to the bicyclist distracted from other roadway considerations to the detriment of safe driving and/or resulted in a tendency to drive toward the bicyclist.

Counter to our hypotheses, we did not observe a link between arousal and attention as measured via eye movements. It does not seem to be the case that individual differences in bicyclist-evoked arousal gave rise to greater attention to the bicyclist, as might have been predicted from studies linking the severity of anxiety concerning a stimulus to attentional biases for that stimulus (e.g., Gerdes et al., 2008; Mogg & Bradley, 2006; Okon-Singer et al., 2011; Vrijsen et al., 2009). There was some evidence that self-reported anxiety (hold-up pressure) was related to attentional processing of the bicyclist, although no relationship was observed using physiological indices. Therefore, although elevated bicyclist-induced arousal and attentional processing of the bicyclist were both associated with safe driving, it does not seem to be the case that one led to the other but rather that each exerts a distinct influence on driving behavior.

Overtaking is a complex set of behaviors with multiple phases, and work remains to be done to truly understand just what are safe interaction behaviors and what are the best proxies for safe interactions. Caution should be taken in interpreting whether the observed variation in driving behavior that included maintaining a greater distance from the bicyclist and being less likely to pass ahead of a bicyclist is in fact a reflection of *safer* driving, although they do suggest more explicitly cautious driving in the presence of a bicyclist. Another limitation of the present study concerns the context in which attention to the bicyclist can be interpreted. We use attention to the bicyclist as an individual differences measure, taken as a proportion of all available gaze data (and thereby reflecting the overall priority afforded to the bicyclist). It is impossible to know to what degree such attention was strategic or involuntary. A more purely involuntary measure of attention to the bicyclist might be more closely linked to physiological arousal and can only be assessed in an experiment in which a suitable comparison stimulus can be used as a basis for comparison to the bicyclist. Such an experiment would likely need to include portions of the drive in which bicyclists are in view but legitimately irrelevant to the task, precluding a measure of safe driving during driver–bicyclist interaction (which necessarily renders the bicyclist task-relevant) which was the focus of the present study.

In the present study, we measured heart rate, breathing rate, and EDA as indicators of bicyclist-evoked arousal. Prior research involving this sample of participants indicated self-reported feelings of nervous and being startled by a bicyclist’s presence (Goddard et al., 2020). From this and related research on how drivers tend to perceive interactions with bicyclists, e.g., Johnson et al., 2014; Goddard et al., 2016; Goddard, 2017), we interpret this arousal as negatively valenced. However, the specific locus of the physiological arousal measured in the present study could reflect a number of interrelated factors, including emotional responses to the bicyclist, anxiety about navigating the bicyclist interaction while being observed in the simulator, and motivation to perform well in anticipation of what might have been perceived as a more difficult upcoming maneuver and the associated cognitive workload involved in preparing for this maneuver. Although the mean changes in physiological arousal linked to the bicyclist may seem numerically small, these changes are comparable to changes linked to viewing more intuitively arousing stimuli, including threatening video clips and erotica (e.g., Fanti et al., 2017; Palomba et al., 2000). Given the complexion of the observed correlations, it does not appear to be the case that physiological arousal was synonymous with self-reported anxiety concerning bicyclists more generally, although it is important to note that our self-report measures draw on reports concerning life experience outside of the simulator environment and so reflect a fundamentally different measure of reactivity.

### Conclusions and implications

Our findings offer a window into how the degree of stimulus-evoked arousal and attentional bias affect goal-directed behavior in a real-world situation. We see evidence that stronger physiological arousal evoked by bicyclists and greater attention to bicyclists (as measured from saccades and fixations) are each associated with safer driving behavior, consistent with the facilitation of goal-directed behavior. Counter to our hypothesis, however, attention and arousal were themselves unrelated; it does not seem to be the case that physiological arousal was responsible for variation in attention in our task. We found evidence that self-reported anxiety concerning driver–bicyclist interactions encountered in everyday life was positively correlated with attention to bicyclists in our task, although greater self-reported anxiety was not itself associated with safer driving. Interestingly, the speed of initial orienting was predictive of the sustained attention measures (fixation duration and number of saccades to the bicyclist) which were themselves predictive of safe driving; this highlights the potential value of quickly recognizing and attending to bicyclists.

One implication of our findings is that efforts to reduce driver anxiety concerning bicyclists should be taken with caution; although at certain levels, bicyclist-evoked anxiety and arousal may become maladaptive (e.g., Etkin & Schatzberg, 2012; Etkin et al., 2010; Krug & Carter, 2012; Yerkes & Dodson, 1908), at least at mild-to-moderate levels, bicyclist-evoked arousal may facilitate safer driving by promoting more vigilant behavior. Likewise, we do not see evidence of over-attending to bicyclists serving as a detriment to safe driving. As with bicyclist-evoked anxiety, there may be a point at which attending to a bicyclist while driving becomes detrimental to driving performance, although the present study suggests that the prospect of over-attending is unlikely to be a salient concern when it comes to promoting safe driver–bicyclist interactions. Finally, our findings suggest that attention and physiological arousal can separately influence driving behavior, casting some measure of doubt on the utility of interventions designed to influence one by manipulating the other.

## Data Availability

The datasets used and/or analyzed during the current study are available from the corresponding author on reasonable request. The datasets used and/or analyzed during the current study are available from the corresponding author on reasonable request. No experiments in this study were preregistered.
